# High Resolution Melting Analysis: A Rapid and Accurate Method to Detect *CALR* Mutations

**DOI:** 10.1371/journal.pone.0103511

**Published:** 2014-07-28

**Authors:** Cristina Bilbao-Sieyro, Guillermo Santana, Melania Moreno, Laura Torres, Gonzalo Santana-Lopez, Carlos Rodriguez-Medina, María Perera, Beatriz Bellosillo, Silvia de la Iglesia, Teresa Molero, Maria Teresa Gomez-Casares

**Affiliations:** 1 Hematology Department, Hospital Universitario de Gran Canaria Dr. Negrin, Las Palmas de Gran Canaria, Spain; 2 Morfology Department, Universidad de Las Palmas de Gran Canaria, Las Palmas de Gran Canaria, Spain; 3 Preventive Medicine Department, Hospital Universitario de Gran Canaria Dr. Negrin, Las Palmas de Gran Canaria, Spain; 4 Laboratori de Biologia Molecular Servei de Patologia, Hospital del Mar, Barcelona, Spain; 5 Medical Sciences Department, Universidad de Las Palmas de Gran Canaria, Las Palmas de Gran Canaria, Spain; Gentofte University Hospital, Denmark

## Abstract

**Background:**

The recent discovery of *CALR* mutations in essential thrombocythemia (ET) and primary myelofibrosis (PMF) patients without *JAK2/MPL* mutations has emerged as a relevant finding for the molecular diagnosis of these myeloproliferative neoplasms (MPN). We tested the feasibility of high-resolution melting (HRM) as a screening method for rapid detection of *CALR* mutations.

**Methods:**

*CALR* was studied in wild-type *JAK2/MPL* patients including 34 ET, 21 persistent thrombocytosis suggestive of MPN and 98 suspected secondary thrombocytosis. *CALR* mutation analysis was performed through HRM and Sanger sequencing. We compared clinical features of *CALR*-mutated *versus* 45 *JAK2/MPL*-mutated subjects in ET.

**Results:**

Nineteen samples showed distinct HRM patterns from wild-type. Of them, 18 were mutations and one a polymorphism as confirmed by direct sequencing. *CALR* mutations were present in 44% of ET (15/34), 14% of persistent thrombocytosis suggestive of MPN (3/21) and none of the secondary thrombocytosis (0/98). Of the 18 mutants, 9 were 52 bp deletions, 8 were 5 bp insertions and other was a complex mutation with insertion/deletion. No mutations were found after sequencing analysis of 45 samples displaying wild-type HRM curves. HRM technique was reproducible, no false positive or negative were detected and the limit of detection was of 3%.

**Conclusions:**

This study establishes a sensitive, reliable and rapid HRM method to screen for the presence of *CALR* mutations.

## Introduction

Philadelphia-negative myeloproliferative neoplasms include polycythemia vera (PV), essential thrombocythemia (ET) and primary myelofibrosis (PMF) [1]. Among them, *JAK2* mutations are present in 50 to 60% of patients with ET or PMF. In addition, mutations in *MPL* gene are present in 5 to 10% of ET and PMF patients with nonmutated *JAK2* [2]. Very recently, exome sequencing techniques have allowed the identification of somatic mutations at exon 9 of calreticulin gene (*CALR*) in 56%–88% of *JAK2*/*MPL*-unmutated ET and PMF [3], [4].

Calreticulin is a highly conserved endoplasmic reticulum (ER) Ca+^2^ binding chaperone that contributes to glycoprotein folding, however, it is also found in the nucleus, cell membranes and extracellular matrix, and has been related to calcium homeostasis, cell adhesion and immune response [5]. So far, all *CALR* mutations seen in MPN involve exon 9, which encodes the carboxyl terminal C-domain. The C-terminal region contains an acidic domain which allows Ca+^2^ binding and a KDEL signal that is an ER retention motif. Almost all the mutations are insertions and deletions that result in a shift of one base pair in the reading frame. Most of the mutants correspond to a 52 bp deletion (L367fs*46) and a 5 bp TTGTC insertion (K385fs*47) [3], [4], [6], [7]. These frameshift mutations instead of generating a premature protein termination, produce a significantly altered C-terminal domain with still unpredicted consequences [4].

The identification of *JAK2/MPL* mutations has been essential for the diagnosis of myeloproliferative neoplasms, however, around 30–45% of ET and PMF remain wild-type for *JAK2/MPL*, and, in some this cases, diagnosis is difficult. Therefore, the discovery in this subgroup of high incidence of somatic *CALR* mutations that are mutually exclusive with JAK/MPL may provide a new tool in molecular diagnosis of myeloprolipherative neoplasms and may have implications for clinical management of patients.

The detection of *CALR* mutations in most of the published reports is done through PCR followed by Sanger sequencing which is one of the gold standard techniques for mutation detection [3], [4], [6], [7]. However, dideoxy sequencing is expensive, time consuming and sensitivity could be compromised in samples with low mutant rate. Therefore, there is a need to develop more generally applicable, sensitive assays to detect *CALR* mutations for use in diagnostic setting. High-resolution melting (HRM) analysis has been widely introduced in the clinical laboratory due to its simplicity, low cost and rapidity among other features. In this work we study the feasibility of using HRM for the detection of *CALR* mutation in a series of patients with essential thrombocythemias and persistent thrombocytosis without mutations in *JAK2* and *MPL*.

## Methods

### Patients and samples

This retrospective study was carried out in patients diagnosed at the Hematology Department of the Hospital Universitario de Gran Canaria Dr. Negrín. *CALR* analysis was performed in patients with wild-type *JAK2/MPL* including 34 ET diagnosed according the 2008 WHO criteria [1], 21 persistent thrombocytosis suggestive of MPN and 98 suspected secondary thrombocytosis. Clinical characteristics of ET patients with *CALR* mutations were compared to a group of 45 *JAK/MPL*-mutated ET subjects.

The patients gave written informed consent and the study was approved by the ethics committee of the Hospital Universitario de Gran Canaria Dr. Negrín. Genomic DNA was extracted from bone marrow and peripheral blood samples. DNA was obtained out of 400 µL of bone marrow/blood in the MagNA Pure Compact Instrument (Roche Diagnostics GmbH, Mannheim, Germany). The quality and concentration of DNA was assessed with a NanoVue Plus Spectrophotometer (GE Healthcare Bio-Sciences AB, Uppsala, Sweden).

### High-resolution melting analysis of *CALR* mutations

The HRM assays were performed using the LightCycler 480 instrument (Roche Diagnostics, Roche Instrument Center AG, Rotkreuz, Switzerland). All samples were tested in duplicate and two wild-type DNA controls and one positive control were included in each experiment. Twenty nanograms of DNA were amplified in a final volume of 10 µL containing 1X LightCycler 480 High Resolution Melting PCR Master Mix (Roche) which includes as saturating fluorescent DNA-binding dye the LightCycler 480 ResoLight Dye, 0.2 µM of each primer and 2.5 mM MgCl_2_. Primer sequences were as previously published (Forward: 5′GGCAAGGCCCTGAGGTGT3′ and Reverse: 5′GGCCTCAGTCCAGCCCTG3′) and the amplicon size was of 265 bp [3]. LC480 cycling parameters were: initial denaturation at 95°C for 10 min; 45 cycles of 95°C for 10 seconds, 58°C for 10 seconds and 72°C for 20 seconds. The final melting program was denature at 95°C for 1 minute, renaturation at 45°C for 1 minute and melting from 60°C to 95°C, with a ramp of 0.02°C per second and 25 fluorescence acquisitions per degree centigrade.

Results were analyzed as fluorescence *versus* temperature graphs by Gene Scanning software version 1.5.0.39 (Roche Instrument Center, Rotkreuz, Switzerland) with normalized, temperature-shifted melting curves displayed as difference plot. Wild-type and mutated samples were defined as positive and negative controls in the software. Melting curve analysis comprised: normalization of melting curves equaling to 100% initial fluorescence and to 0% the fluorescence remnant; shifting of the temperature axis of the normalized melting curves to the point where the entire double-stranded DNA is completely denatured; and finally, the difference plot analyzing the differences in melting curve shape clustering the samples into groups based on the internal software calculation [8], [9].

### Sequencing

Sanger sequencing was performed on the same amplicons as used for HRM analysis. PCR products were diluted 1∶10 in water and 5 µl of dilution were cleaned with ExoSap-IT (USB Corporation, Cleveland, OH). Bidirectional sequencing reaction was set up with the BigDye Terminator, version 3.1, Cycle Sequencing Kit (Applied Boisystems, Foster City, CA). Performa DTR Gel Filtration Cartridges (Edge Bio, Gaithersburg, MD) were used to purify the reactions. Sequencing analyses were carried out on the on the ABI PRISM 3130 Genetic Analyzer (Applied Biosystems, Foster City, CA).

### Statistical analysis

For parametric and nonparametric continuous variables the Student t test and Mann–Whitney U test were used respectively. Significance was set at a p-value of 0.05 (SPSS statistical package v. 15.0).

## Results

### High-Resolution Melting Analysis

**Figure 1:**
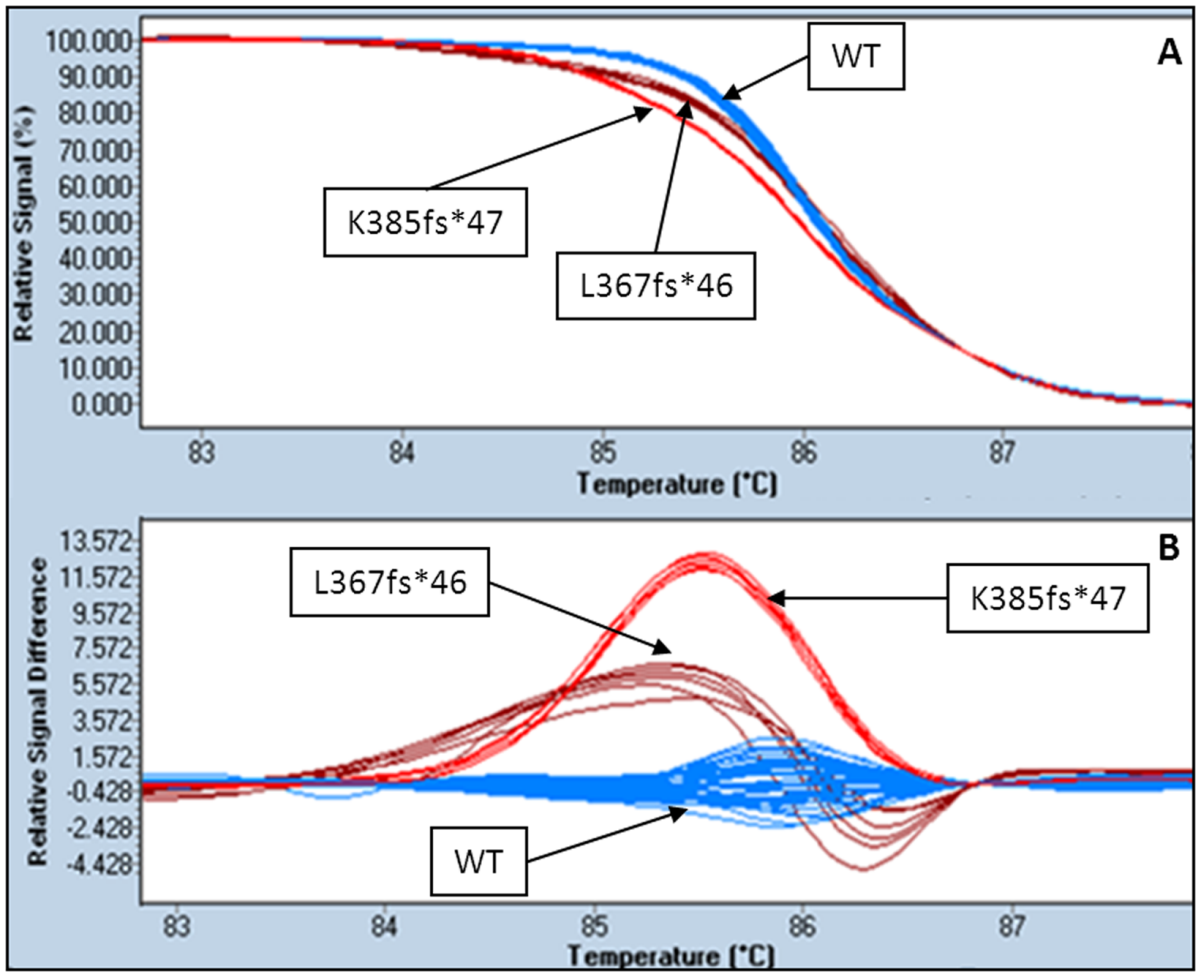
HRM method. Examples of melting curves (A) and difference plots (B) obtained for wild-type (WT) and L367fs*46 and K385fs*47 mutations of *CALR*.

After normalization and temperature shifting, a clear difference was evident between the wild-type group and the rest of the samples. This difference was emphasized by using the fluorescence difference plots, where the curves for the wild-type samples were clustered around the baseline. Among *JAK2/MPL*-negative samples a total of 15 ET and 4 persistent thrombocytosis suggestive of MPN showed a clear distinctive pattern of the melting and difference curve plot compared with those of the wild-type (Figure 1). Moreover, three different patterns could be clearly distinguished among the positive samples.

No positives were found among the 98 secondary thrombocytosis.

### Correlation between HRM and sequencing results. Characterization of mutations

Samples that were positive in HRM analyses were bidirectionally sequenced by using the same PCR product (Figure S1). Of the 15 ET with HRM positive curves, 7 were 52 bp deletions (L367fs*46), another 7 were 5 bp TTGTC insertions (K385fs*47), and one patient showed a complex mutation consisting of an insertion of TTTGTC and a deletion of a T resulting in a K385fs*47 mutant protein.

Of the 4 persistent thrombocytosis suggestive of MPN with positive HRM curves, one was a single nucleotide polymorphism, rs148604761 (c.1245C>T), 2 were L367fs*46 mutations and the other was a K385fs*47 mutation. This latter sample although showing a clear pattern of 5 bp insertion by HRM, the sequence evidenced low mutant allele burden (figure S1).

Deletions of 52 bp and insertions of 5 bp showed clearly distinct HRM patterns, the HRM curve of the complex mutation was similar to the 5 bp insertion (Figure 1 and Figure S2).

Overall, the incidence of *CALR* mutations in wild-type *JAK2* and *MPL* patients was of 44% (15/34) in ET and of 14% (3/21) in persistent thrombocytosis suggestive of MPN.

Samples slightly differing from the wild-type control as well as 45 samples showing a wild-type pattern were also sequenced. None of patients evidenced mutations, even those with a borderline pattern. Thus, no false positive or negative results were found.

### Limit of detection of HRM

**Figure 2:**
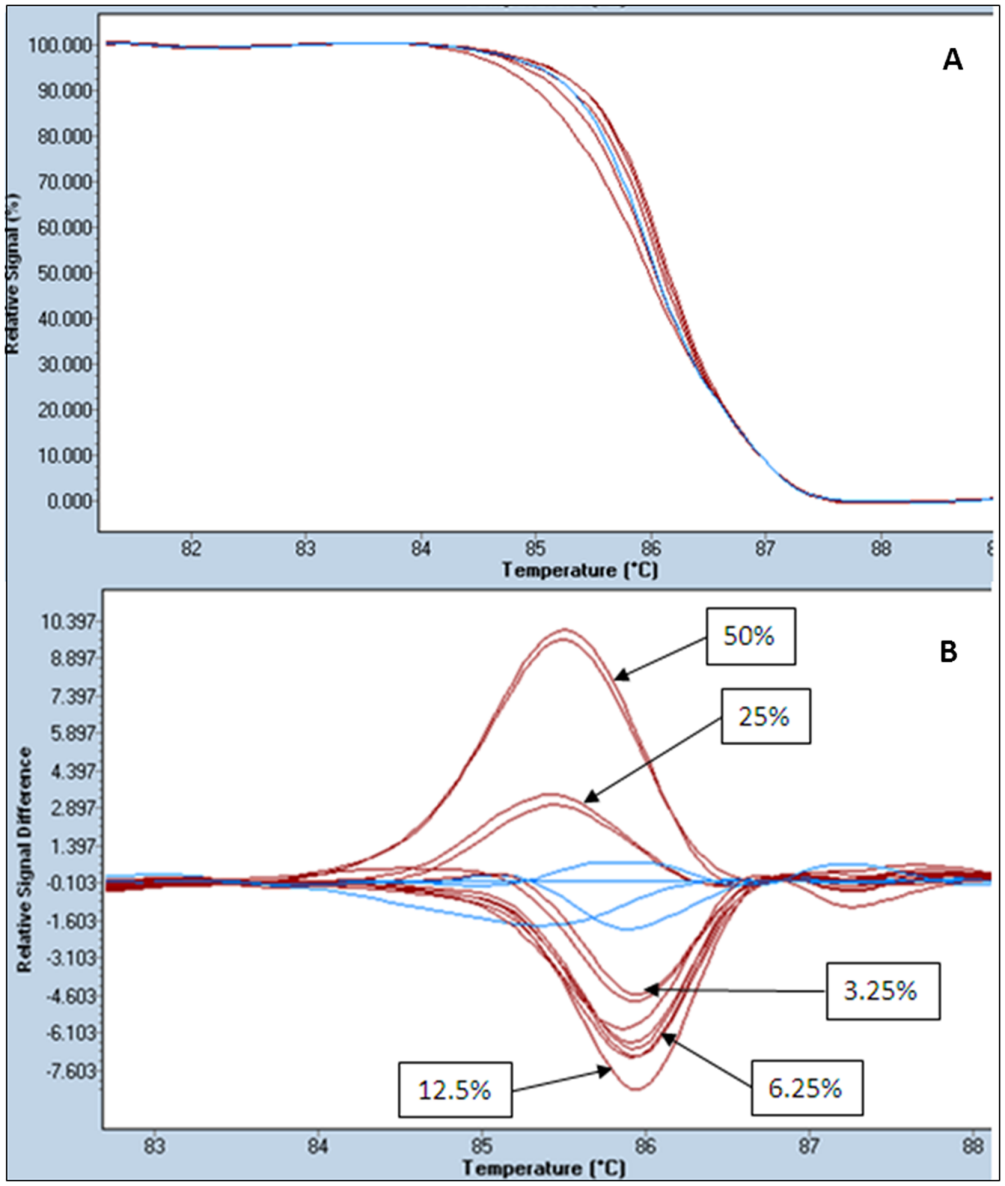
Limit of detection of HRM assay. Melting curves (A) and difference plots (B) of wild-type (blue) and serial dilutions of a *CALR* K385fs*47 mutant (brown).

To determine the sensitivity of the technique, we serially diluted a sample displaying a mutant allele burden of approximately 50% according to sequencing analysis. Serial dilutions were made up to 1.6% of mutant in wild-type DNA. The *CALR* mutant could be detected in up to 3.13% dilution (Figure 2B).

### *CALR* mutations and clinical characteristics of patients

We compared clinical characteristics of the 15 ET *CALR*-mutated samples *versus* 45 ET *JAK*-mutated patients. The mean hemoglobin level was 14.9 g/L lower in *CALR*-positive (130.9 g/L) compared to *JAK2*-positive (145.8 g/L) patients (95% confidence interval 4.8–25.0 g/L, p = 0.004, t-test). The median platelet count was significantly different in *CALR*-mutated (937.0×10^9^/L) *versus JAK2*-mutated patients (713.5×10^9^/L) (p = 0.001, Mann-Whitney). Also, patients carrying *CALR* mutations were younger, although this association was not statistically significant (median 52.0 years for *CALR*+ and 63.5 years for *JAK2*+, p = 0.146, Mann-Whitney).

## Discussion

*CALR* mutations have been recently identified in a significant proportion of patients with thrombocythemia and myelofibrosis without *JAK2* and *MPL* mutations [3], [4]. Hence, is expected that the identification of these new mutations may contribute to the diagnosis of these myeloproliferative neoplasms.

In the majority of the published studies, mutation analysis of *CALR* is done through PCR followed by Sanger sequencing [3], [4], [6], [7]. A recent report has proposed PCR followed by fragment analysis as a screening method as *CALR* mutations are mainly insertions and deletions [10]. We show that the established HRM method allows the identification of exon 9 *CALR* mutations, since the differential plot clearly discriminates mutant from wild-type samples. Samples showing a melting behavior differing from wild-type were shown to have mutations by direct sequencing of HRM PCR products, and none of the samples with wild-type or borderline plot evidenced sequence alterations. No false positives or negatives were observed and we determined the limit of detection of 3% of mutated DNA in wild-type background, whereas the sensitivity of Sanger sequencing is reported as about 15–20% [11]. In fact, in the present work, one of the samples with a clear HRM pattern of 5 bp insertion displayed a low mutant allele burden in the sequence which could have been missed by only sequencing. Therefore, the method presented is reliable, specific and sensitive. The difference plot pattern of the 52 bp deletion was clearly different from the 5 bp insertion. However, since other 5 bp insertions different from the common TTGTC have been described and even complex mutations, as the one we have found, can result in a 5 bp insertion, sequencing is necessary to correctly categorize the mutant.

*CALR* mutational frequency in our series of *JAK2/MPL*-unmutated ET was of 44%, which was similar to that obtained by Rotunno et al. [6] and significantly lower compared to others (67–71%) [3], [4]. As has been pointed, this discrepancy could be due to differences in diagnostic criteria since Rotunno et al. used strict WHO criteria, whereas the others did not and might have included patients with early/prefibrotic PMF [12].

Most of the described mutations are insertions/deletions, and 52 bp deletion (L367fs*46) and TTGTC insertion (K385fs*47) constitute around 85% of reported *CALR* mutations [3], [4], [6], [7], [10], [12]. In the present study 50% of the patients showed 52 bp deletions, 44.4% of the subjects displayed the 5 bp deletion, one patient had a complex mutation and a single nucleotide polymorphism was identified in other. According to what has been described, these frameshift mutations within exon 9 of *CALR*, instead of generating a premature protein termination, produce a significantly altered C-terminal domain with still unknown consequences [4]. It has been suggested that *CALR* mutations may, as well as *JAK2* mutations, activate the JAK-STAT signaling pathway, which would explain the mutually exclusive presence of mutations [3], [13]. Nevertheless, the mechanism remains unclear.

As has been reported in ET, we found that *CALR versus JAK2* mutations were significantly associated with lower hemoglobin level and higher platelet count and, although not significant, probably due to the limited series, to younger age at diagnosis [6], [7]. Also, *CALR* mutants have been reported to present superior thrombosis-free survival [6], [7] and, according to other study, better overall survival [3].

According to what has been proposed, *CALR* mutations could be used as a diagnostic tool in the same way *JAK2* alterations have improved accuracy of current diagnosis of essential thrombocythemia and primary myelofibrosis. In this sense *CALR* mutations may be a useful addition to the WHO criteria for these disorders. Thus, here we propose HRM as a reliable, economical, fast, sensitive and high-throughput screening method, which would precisely select samples for mutation characterization through direct sequencing or quantification of mutated allele by fragment analysis.

## Supporting Information

Figure S1 **Sanger sequencing confirmation of positive HRM samples.** CALR reverse strand of a wild type sample (A), a L367fs*46 mutant (B) and a K385fs*47 patient with low mutant allele burden (C).
(TIF)

Figure S2 **Melting curves (A) and difference plots (B) obtained for wild-type (WT), positive control (C+) and the single nucleotide polymorphism (rs148604761).**
(TIF)
