# Supplementary figures and images for: High Resolution Melting Analysis: A Rapid and Accurate Method to Detect CALR Mutations
Source: PLoS One. 2014 Jul 28;9(7):e103511. doi: 10.1371/journal.pone.0103511 (PMC4113452; doi:10.1371/journal.pone.0103511)

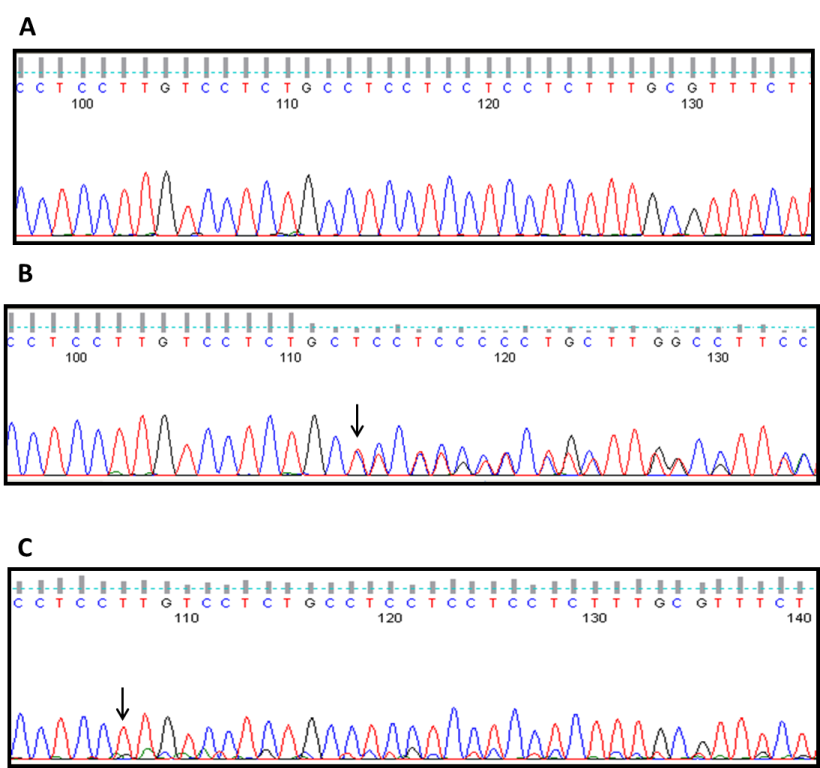

Supplement: Figure S1 — Sanger sequencing confirmation of positive HRM samples. CALR reverse strand of a wild type sample (A), a L367fs*46 mutant (B) and a K385fs*47 patient with low mutant allele burden (C). (TIF) [file pone.0103511.s001.tif]

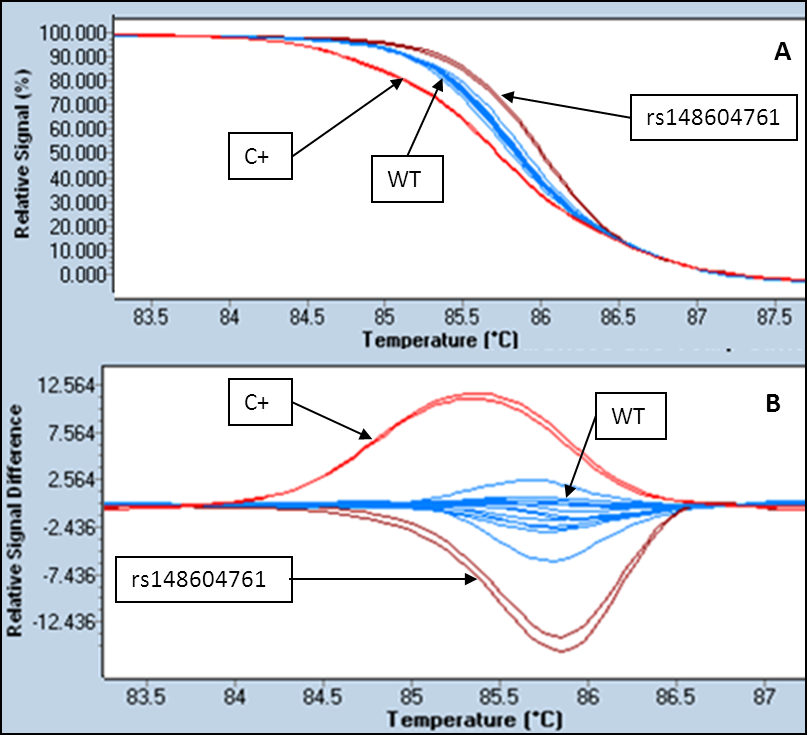

Supplement: Figure S2 — Melting curves (A) and difference plots (B) obtained for wild-type (WT), positive control (C+) and the single nucleotide polymorphism (rs148604761). (TIF) [file pone.0103511.s002.tif]
